# Sodium Starch Glycolate (SSG) from Sago Starch (*Metroxylon sago*) as a Superdisintegrant: Synthesis and Characterization

**DOI:** 10.3390/molecules29010151

**Published:** 2023-12-26

**Authors:** Okta Nama Putra, Ida Musfiroh, Sarah Elisa, Musa Musa, Emmy Hainida Khairul Ikram, Chaidir Chaidir, Muchtaridi Muchtaridi

**Affiliations:** 1 Department of Pharmaceutical Analysis and Medicinal Chemistry, Faculty of Pharmacy, Padjadjaran University, Jatinangor 45363, West Java, Indonesia; okta22001@mail.unpad.ac.id (O.N.P.); ida.musfiroh@unpad.ac.id (I.M.); 2 Research Centre for Agroindustry, National Research and Innovation Agency (BRIN), Cibinong 16912, West Java, Indonesia; sara003@brin.go.id (S.E.); musa002@brin.go.id (M.M.); 3 Centre for Dietetics Studies and Integrated Nutrition Science and Therapy Research Group (INSPIRE), Faculty of Health Sciences, Universiti Teknologi MARA Cawangan Selangor Kampus Puncak Alam, Bandar Puncak Alam 42300, Selangor, Malaysia; emmy4546@uitm.edu.my; 4 Research Center for Pharmaceutical Ingredient and Traditional Medicine, National Research and Innovation Agency, Cibinong 16912, West Java, Indonesia; chai001@brin.go.id; 5 Research Collaboration Centre for Radiopharmaceuticals Theranostic, National Research and Innovation Agency (BRIN), Jl. Soekarno KM-21, Sumedang 45363, West Java, Indonesia

**Keywords:** sodium starch glycolate (SSG), sago starch, cross-linking, degree of substitution (DS), superdisintegrant

## Abstract

The characteristics of sago starch exhibit remarkable resemblances to those of cassava, potato, and maize starches. This review intends to discuss and summarize the synthesis and characterization of sodium starch glycolate (SSG) from sago starch as a superdisintegrant from published journals using keywords in PubMed, Scopus, and ScienceDirect databases by Preferred Reporting Items for Systematic Reviews and Meta-Analyses (PRISMA 2020). There are many methods for synthesizing sodium starch glycolate (SSG). Other methods may include the aqueous, extrusion, organic solvent slurry, and dry methods. Sago starch is a novel form of high-yield starch with significant development potential. After cross-linking, the phosphorus content of sago starch increases by approximately 0.3 mg/g, corresponding to approximately one phosphate ester group per 500 anhydroglucose units. The degree of substitution (DS) of sodium starch glycolate (SSG) from sago ranges from 0.25 to 0.30; in drug formulations, sodium starch glycolate (SSG) from sago ranges from 2% to 8% *w*/*w*. Higher levels of sodium starch glycolate (SSG) (2% and 4% *w*/*w*) resulted in shorter disintegration times (within 1 min). Sago starch is more swellable and less enzymatically digestible than pea and corn starch. These investigations demonstrate that sago starch is a novel form of high-yield starch with tremendous potential for novel development as superdisintegrant tablets and capsules.

## 1. Introduction

Starch is a biodegradable polymer that is economical and derived from renewable resources. Consequently, it is suitable as a primary material in the food, textile, pharmaceutical, and medical industries [1]. Starch is extensively used as a versatile excipient in many solid dosage forms, primarily because of its favorable physicochemical characteristics, cost-effectiveness, and inert nature. It is frequently used in application as a diluent, disintegrant, and binder in tablet formulations [2]. However, native starch is unstable under processing conditions, including temperature, shear, and pH changes, which limits its applications. Physicochemical and enzymatic modifications often cultivate desired functional characteristics, including enhanced adhesion, solubility, and heat resistance [3]. Starch modification involves different cases, such as using the a-amylase enzyme. Treating granules with enzymes significantly impacted those containing more amorphous components (lower crystallinity) [4]. Elahe Abedi’s report indicates that the starch’s enzymatic digestibility increased, resulting in significant morphological alterations, including the formation of pores and outgrowth.

Additionally, the starch’s crystalline structure became disorganized, leading to a decrease in its swelling power. The hydrolyzed St-Cys conjugates exhibited reduced peak and final viscosity and lower pasting and gelatinization temperatures. A deeper understanding of breaking down starch in industrial settings can decrease energy usage and costs by optimizing efficiency [5,6]. The versatility of starches means that new starch excipients with suitable properties must be further developed to meet the specific needs of pharmaceutical formulators and the needs of new formulations.

Different fractions of polysaccharides, such as amylose and amylopectin, build up starch. The structure of these polysaccharides consists of chains of glucose molecules that exhibit variability in both size and form. Amylose comprises glycosidic α(1,4) linkages, which give rise to a linear helical conformation. Amylopectin has a significantly branched semi-crystalline configuration, characterized by the presence of α(1,4)-glycosidic bonds and 4–5% α(1,6)-glycosidic bonds, which are accountable for its branching architecture. Amylopectin and amylose assemble as a semi-crystalline complex with the formation of hydrogen bonds between the double helices, which are mainly composed of branched amylopectin chains.

Sago starch has considerable promise as a source of natural macromolecules. Indonesia is among the leading global sago producers, behind Malaysia and Papua New Guinea. Sago starch has a comparatively lower cost in comparison to other starches. However, the characteristics of sago starch exhibit striking similarities to those of cassava, maize, and potato [7]. Sago starch is more swellable and less enzymatically digestible than pela and corn starch [8]. Sago starch may be used as a stabilizer since it has a greater gelatinization temperature and lower peak viscosity than other types of starch. Sago starch has also produced traditional delicacies such as pasta, pastries, cakes, and bread. In addition, sago starch is crucial to the textile, paper, and pharmaceutical industries [9].

Sago starch is innovative with a high-yield characteristic and holds much promise for further development. However, in contrast to commonly used carbohydrates such as potato and maize starch, sago starch has not been widely adopted owing to a lack of comprehensive study on its qualities. This study comprehensively analyzes sago starch’s morphology, amylose concentration, crystal structure, pasting capabilities, and hydrolysis properties [10].

Tablets are often produced by the fast compression of multicomponent powder portions that consist of the active ingredient and excipients [11]. Tablets must have certain qualities, including quality, friability, disintegration, and physical consistency. The demand for such precise dosage forms has created many excipients that bestow particular tablet properties [12].

A disintegrant is a compound or combination of compounds used in a pharmaceutical formulation to disperse or fragment a tablet or capsule’s constituents, promoting expeditious disintegration [13]. Superdisintegrants are compounds that aid in the rapid disintegration of pharmaceutical formulations with a reduced amount compared to regular disintegrants [14]. The disintegration profile of pharmaceutical tablets may be modified by incorporating “super-disintegrants”. Polymer-based compounds, including sodium starch glycolate (SSG), croscarmellose sodium, and crospovidone, exhibit volumetric expansion upon exposure to water, resulting in fast disintegration of the tablet matrix [15].

A chemically altered starch known as SSG, or sodium starch glycolate, is frequently used to disintegrate pharmaceutical capsules and tablets. Sodium starch glycolate (SSG) may be characterized as the sodium salt derived from the carboxymethyl ether of starch. Two chemical modification techniques often produce Starch Sodium Octenyl Succinate (sodium starch glycolate—SSG) from starch. The first technique is substitution, which aims to enhance the hydrophilicity of the starch. The second technique is cross-linking, which decreases gel formation and solubility when the modified starch comes into contact with water [16,17].

Several manufacturers, such as Primojel, Explotab, and Vivostar, offer this material under brand names. The constituents for Primojel and Explotab are produced by reacting potato starch with sodium chloroacetate. However, whether the potato starch is pre- or post-substitution is not apparent. Vivastar P cross-links the material through Na carboxylic acid groups and starch alcohol groups following substitution [18].

Sodium starch glycolate may be described as the sodium salt derivative of carboxymethyl ether. Starch glycolate is obtained from several sources, such as rice, potatoes, wheat, or maize. Sodium Starch Glycolate is a white to off-white powder with a light odor and exhibits moderate free-flowing properties [19]. Sodium starch glycolate is a pharmaceutical excipient that enhances drug solubility for both tablets and capsules throughout the drug evaluation process. Sodium starch glycolate has a quick moisture absorption property, resulting in swelling and subsequent expedited breakdown of tablets and granules. The substance in question is used in several capacities within the field, including but not limited to its function as a disintegrant, suspending agent, and gelling agent [20]. In the absence of a disintegrant, the dissolution process of the tablet may be compromised, potentially impacting the active component’s bioavailability. This particular substance may be found under many commercial designations from various manufacturers. Three often used excipients in the pharmaceutical industry are Explotab, Primojel, and Vivastar [21].

Indonesia has a potential sago land of 5.5 million hectares. However, its utilization has only reached 5%. Thus, the development potential of sago continues to expand to all regions of Indonesia [21]. Developing and applying sago starch as superdisintegrant tablets or capsules is a novelty.

### Structure

**Figure 1:**
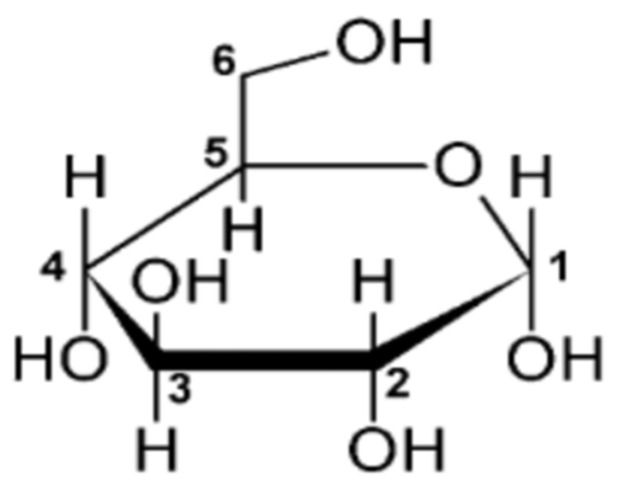
The chemical structure of glucose.

The α- or β- form of glucose is determined by the location of the hydroxyl group at position 1, as seen in Figure 1.

**Figure 2:**
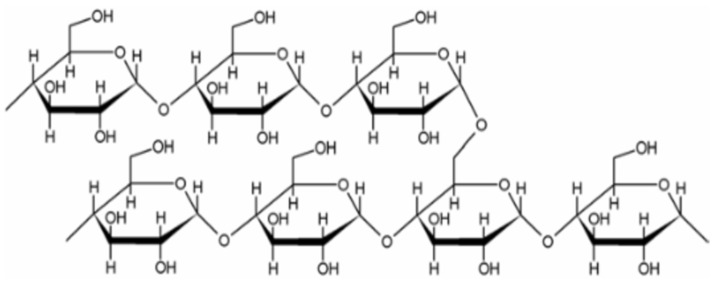
The chemical composition of a specific segment of amylopectin.

Starch is composed of two α-glucose polymers: Sago starch is composed of two different types of amyloses, a linear polymer of -glucose joined at carbons 1 and 4, and amylopectin, a branched polymer of glucose, which consists of shorter straight chains that extend across the central region. The polymer consists of repeating units, namely carbon atoms 1 and 4, and an extra link connecting carbon atoms 1 and 6. The typical degree of polymerization is 2,000,000. Sago starch contains about 24.07–26.11% amylose and 73.89–75.93% amylopectin [10]. A portion of an amylopectin molecule showing 1,4 and 1,6 linkages is shown in Figure 2.

## 2. Materials and Methods

- Literature search

The research methodology used in this study was guided by the Preferred Reporting Items for Systematic Reviews and Meta-Analyses (PRISMA 2020) guidelines [22]. The objective of the literature search conducted in this systematic review is to discover pertinent papers about the potential of sodium starch glycolate. We comprehensively selected electronic databases such as PubMed, Scopus, and ScienceDirect. These keys include (1) sodium starch glycolate and (2) potato or sago starch.

- Inclusion criteria

Only research discussing the potential of sago starch, cross-linking and synthesis, physical and chemical properties, and functional properties of tablets as disintegrants were selected for further analysis. The selected articles must be written in English. The articles selected must evaluate at least the following: (1) sago starch, (2) cross-linking and synthesis, (3) physicochemical properties, and (4) superdisintegrant.

- Exclusion criteria

The publications excluded from the systematic review include proceedings, theses, dissertations, articles requiring English language composition, papers without titles, abstracts, and keywords that fail to match the predetermined inclusion criteria. This research focuses on the process of synthesis and characterization of sodium starch glycolate as a superdisintegrant.

- Study selection

Three writers selected pertinent publications by thoroughly examining the title, abstract, and keywords. This screening established the eligibility criteria (ONP, SE, and MM). The two authors (MM and IM) were involved in the discussion to decide on possible disagreements between the authors. The whole text of the relevant published article was then examined. The reference management software Mendeley version 1.19.8 and Endnote 20 was used to aggregate the chosen papers for inclusion in this literature review.

- The literature searches

**Figure 3:**
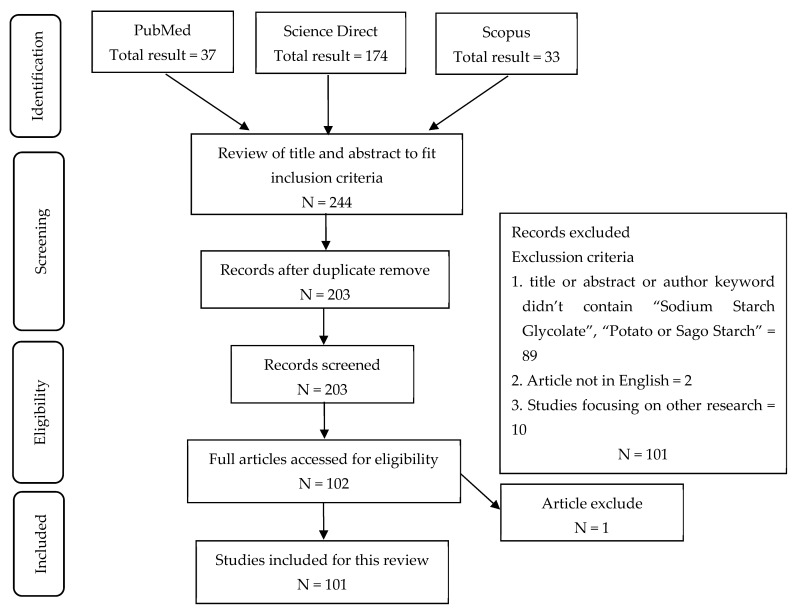
Flow chart showing the literature search.

A comprehensive compilation of 244 articles was discovered, all of which were deemed pertinent to the subject matter at hand. Following the process of duplicate identification, a total of 41 publications were removed from the dataset. One hundred two publications were rejected based on the title, abstract, keywords, and inclusion criteria about sago starch, cross-linking and synthesis, physicochemical qualities, and superdisintegrant. The inability to obtain one report resulted in its exclusion from the comprehensive work. Consequently, a thorough examination of 101 publications was conducted to analyze the systematics, as seen in Figure 3. Data extraction is conducted by selecting finished articles.

### 2.1. The Process of Choosing a Starch Source

Sodium starch glycolate can be synthesized using many types of natural starches (Table 1). Sago starch is a novel variety that exhibits exceptional productivity and holds significant promise for future advancements [10]. Nevertheless, the utilization of sago starch is limited due to inadequate investigation into its characteristics compared to more established starches like potato and maize starch [23]. Sodium potato starch glycolate, derived from potato starch, exhibits the maximum water absorption capacity and leads to the most rapid disintegration when employed with lactose placebo tablets [21]. The primary cause of tablet disintegration of tablets composed of this insoluble substance is mainly ascribed to expansion disintegration and strength enhancement rather than water infiltration. However, the influence of the starch origin on this phenomenon is relatively insignificant. Various sodium starch glycolate (SSG) grades are available, depending on particle size distribution, sodium chloride (NaCl) content, and pH levels [24].

### 2.2. The Substance Known as Sodium Starch Glycolate (SSG) Is Derived from Sago Starch

Sodium starch glycolate (SSG), widely utilized in several industries, can be procured from manufacturers that market it under distinct trade names such as Primojel, Explotab, Glycolys, and Vivastar. The chemicals utilized in producing Primojel and Explotab [25] involve the chemical reaction between potato starch or sago starch and sodium chloroacetate. Vivastar P facilitates cross-linking of the substance using sodium carboxylic acid and starch alcohol groups [26]. Starch glycolate is obtained from several sources, such as rice, potatoes, wheat, or corn [27]. Sodium starch glycolate (SSG), obtained from sago starch, is a chemically modified starch that has undergone cross-linking and substitution. It is commonly employed as a disintegrant in pharmaceuticals [24]. Sodium starch glycolate (SSG) sago may be described as the sodium salt obtained from the carboxymethyl ether of starch. Two chemical modification procedures are commonly employed to create sodium starch glycolate (SSG) (sago starch granules) from sago starch. The first process involves substitution, which aims to enhance the hydrophilicity of the starch. The second process involves cross-linking, which is utilized to decrease gel formation and the starch’s solubility when it comes into contact with water [16]. Table 2 displays the documented procedures involved in the production of sodium starch glycolate (SSG) from different brands.

Following the selection of a suitable source of starch, the subsequent stage in the synthesis process involves the cross-linking of the starch. Using cross-linking polymerization and acetylation techniques leads to the formation of a modified form of cross-linked esterified starch. Cerium (IV) oxide has notable attributes concerning its thermal stability, acid resistance, and resistance to shearing pressures, as documented in a previous study [28]. Cross-linking decreases solubility and promotes gel formation when in contact with water [16]. The process is commonly conducted by employing a starch-esterifying agent approved by the Food and Drug Administration (FDA), such as sodium trimetaphosphate or phosphorus oxychloride, in an alkaline suspension [29]. The formation of starch phosphate occurs through the cross-linking of two components, as depicted in Equations (1) and (2) below [30].

2StOH + Na_3_P_3_O_9_ → StO-P(OONa)-OSt + Na_2_H_2_P_2_O_7_ (1)

2StOH + POCl_3_ + NaOH → St-O-P(OONa)-OSt + 3NaCl + 3H_2_O (2)

The evaluation of cross-linking extent is not assessed in the official monographs; however, it is observed to be minimal. The cross-linking process leads to an approximate increase of 0.3 mg/g in the phosphorus content of sago starch. Utilizing a blend of 12% sodium tripolyphosphate and sodium trimetaphosphate for cross-linking starches has resulted in significant enhancements. The improvements encompass a significant rise in replacement levels, with 0.4% phosphorus bound, a relatively higher overall concentration of dietary fiber ranging from 58% to 76%, and a reduced ability to swell compared to native starches [31].

**Figure 4:**
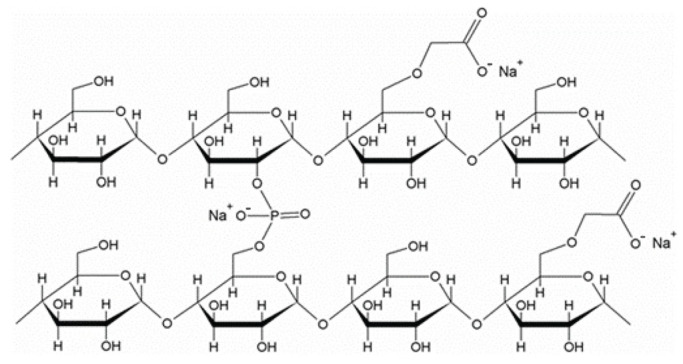
Representative parts of cross-linked and substitution.

Starch cross-linking is achieved by substituting chloroacetic acid or sodium monochloroacetate in an alkaline alcohol suspension, following Williamson’s ether synthesis principles. The replacement of chlorine in sodium chloroacetate is facilitated by the deprotonated starch nucleophile, as evidenced by Equation (3) and Figure 4.

2StO^−^ + ClCH_2_CO_2_Na → StO. CH_2_CO_2_Na + Cl^−^ (3)

Following the completion of the replacement process, the reaction mixture undergoes neutralization, resulting in the isolation and subsequent drying of the sodium starch glycolate. The sodium starch glycolate exhibits a degree of substitution (DS) ranging from 0.23 to 0.32, compared to the theoretical maximum value of 3. This maximum value indicates the complete substitution of all three hydroxy groups in the anhydroglucose units. Hence, around 25% of the anhydroglucose units undergo carboxymethylation. Figure 4 illustrates a segment that is cross-linked with phosphate groups.

## 3. Physical Chemistry of Sago Starch

Sodium starch glycolate is mainly used as a disintegrant [16]. Physical chemistry characterization of sodium starch glycolate includes differences in the degree of substitution (DS), reaction efficiency (RE), sodium chloride (NaCl) analysis, degree of gelatinization (DG) particle size, surface area, pasting properties, bulk and tapped densities, porosity, hydration capacity, water solubility or swelling power, surface morphology, identification of functional groups (Fourier transform infrared—FTIR) and a viscosity [32]. Sodium starch glycolate is a powdered substance that appears white to off-white. It has a light scent, or lack thereof, and exhibits moderate free-flowing properties [33]. The analytical method is described below.

### 3.1. Chemical Analysis of the Degree of Substitution (DS) of Carboxymethyl Starch (CMS)

Following the procedure described in reference [34], the degree of substitution (DS) is calculated. The samples (50 mg) were diluted using concentrated nitric acid (4 cm^3^) in a glass container fitted with a heated plate. Digestion was performed with deionized water up to 100 cm^3^. The study was conducted using a flame atomic absorption spectrometer. The experimental setup included using an air-acetylene flame composition, whereas sodium’s observed wavelength was 589.0 nm. The following procedures were used to calculate the level of substitution:

(4) $$\mathrm{DS}=\frac{162\%\;Na}{\left(2300-80\%\;Na\right)}$$

The unmodified starch’s % sodium (Na) was previously determined and corrected with carboxymethyl starch (CMS) derivatives [35].

The chemical measurement method most commonly employed in the literature was direct titration, which provided a dependable assessment of the degree of substitution (DS) of carboxymethyl starch (CMS) [36]. Therefore, the direct titration method was selected to determine the degree of substitution (DS) of carboxymethyl starch (CMS) in the present investigation. The carboxymethyl starch (CMS) (5 g) was dispersed in a solution of acetone (150 mL), followed by the addition of 5 M HCl (15 mL) to the dispersion. The resulting mixture was agitated for 30 min. Throughout this procedure, the sodium variant of carboxymethyl starch (CMS) underwent a conversion to H-CMS, which stands for carboxymethyl starch (CMS) in its hydrogen form. The H-CMS was subjected to many washes using a solution of 80% (*v*/*v*) methanol until the pH test indicated neutrality. The neutral dispersion was subjected to a second filtration, mixed with acetone, and agitated for 15 min. Subsequently, the mixture was filtered and left to dry for 24 h in a silica gel desiccator. A solution of 1% (*w*/*v*) sodium chloride (NaCl) was prepared and used to dissolve a sample of H-CMS weighing two grams. The solution was titrated using 1 M NaOH [37,38]. The determination of the degree of substitution (DS) was performed in the following manner:

(5) $$m_{c}=m_{p}-\frac{mp\times F}{100}\;\;\mathrm{DS}=\frac{nNaOH\times mo}{mc-nNaOH\times mr}$$

M0 = the molar mass of the anhydroglucose unit is 162 g/mol. The molar mass of the carboxymethyl residue is 58 g/mol. The amount of sodium hydroxide utilized is represented by nNaOH (in mol). The weight of the polymer collected is denoted as mp (in g). The corrected weight of the polymer is represented by mc (in g). The moisture content is denoted as F (%) [39].

### 3.2. Reaction Efficiency (RE)

The proportion of reactants used to determine each experiment’s reaction efficiency (RE) was determined. This was achieved by dividing the observed degree of substitution (DS) by the theoretical degree of substitution (DS) [40]. The quantification of the response efficiency (RE) in each experimental trial is established as follows:

(6) $$\mathrm{RE}\;(\%)=\frac{DSexp}{DSth}\times 100\%$$

DSth is a theoretical degree of substitution (DS), and DSexp is the experimental degree of substitution (DS) obtained after the reaction. The reaction efficiency (RE) percentage for sodium starch glycolate (SSG) is calculated using the following formula.

(7) $$\mathrm{RE}\;(\%)=\frac{w2-w1}{W3\times 100}$$

W1, W2, and W3 are the weights of initial strength, carboxymethyl starch (CMS), and sodium monochloroacetate, respectively. The assessment of reaction efficiency (RE). The determination of reaction efficiencies (RE) was conducted using the following equation, as elucidated by the following [41]:

(8) $$\begin{array}{l} \mathrm{RE}=\frac{DS\times 100}{Dst}\\ \mathrm{where}:\;\mathrm{Dst}\;\frac{nSMCA}{nAGU},\;\mathrm{If}\;\mathrm{nNAOH}\;\geq \;\mathrm{nSMCA}\\ \mathrm{Dst}:\;\frac{nNaOH}{nAGU},\;\mathrm{If}\;\mathrm{nNAOH}\;<\;\mathrm{nSMCA} \end{array}$$

The variables NSMCA, nAGU, and nNaOH represent the number of moles of sodium monochloroacetate, anhydroglucose unit, and sodium hydroxide, respectively.

### 3.3. Sodium Chloride (NaCl) Measurement

During the synthesis process of superdisintegrants, by-product impurities can be generated. The synthesis of sodium starch glycolate (SSG) yields many reaction by-products, including sodium chloride (NaCl), sodium glycolate, and sodium citrate. The official compendia, such as USP 32/NF 27 and PhEur 6.0, have specific specifications for the quantities of by-products present in sodium starch glycolate (SSG). According to these compendia, the sodium chloride (NaCl) concentration should be below 7%, while the sodium glycolate content should be below 2% [42]. Elevated consumption of sodium chloride (NaCl) and increased sodium concentration levels are factors that increase the risk of stroke, thrombosis, cardiac arrhythmias, obesity, diabetes, kidney disease, hepatic disease, multiple sclerosis, systemic sclerosis, migraine, tinnitus, Bell’s palsy, polycystic ovary syndrome, and pneumonia [43]. Consuming excessive salt, exceeding the minimum requirement of 500 mg for healthy bodily function, can lead to sodium poisoning. Consuming natural foods that do not include added sodium chloride (NaCl) can meet this minimum requirement. Sodium chloride (NaCl) comprises around 40% sodium by weight, and the average American consumes an excessive amount of sodium, with an average daily intake of 3300 mg [43].

Sodium chloride (NaCl) concentration in refined carboxymethyl starch (CMS) was determined using a protocol described in the British Pharmacopoeia. A suspension was prepared by combining 0.5 g of pure carboxymethyl starch (CMS) with 1 mL of nitric acid in 100 mL of distilled water. The suspension was then subjected to potentiometric titration using a 0.1 M silver nitrate solution and an indicator electrode. A volume of 1 milliliter of a 0.1 molar solution of silver nitrate may have the exact chemical equivalence as 5.844 milligrams of sodium chloride (NaCl).

The sodium chloride (NaCl) content was determined by the following calculation:

(9) $$\mathrm{NaCl}\;(\%)=\frac{V1\frac{5844}{1000}}{w1}\times 100$$

V1 (mL) represents the volume of 0.1 M silver nitrate solution added until the point of inflection was attained, whereas W1 (g) denotes the mass of carboxymethyl starch (CMS) [44].

### 3.4. Fourier Transform Infrared (FTIR) Spectroscopy

**Figure 5:**
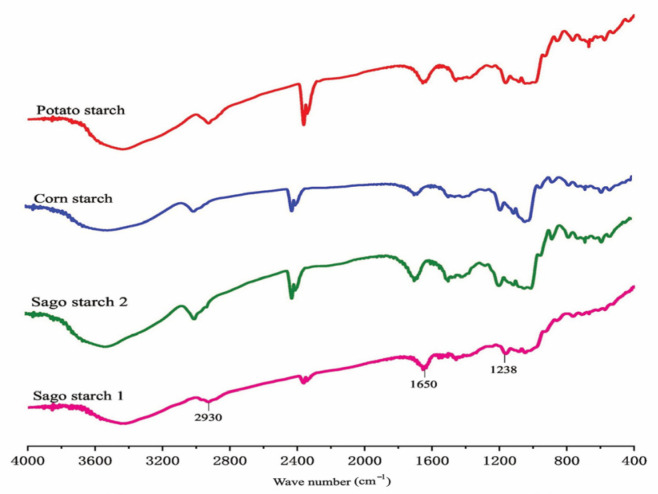
Fourier transform infrared (FTIR) spectra of starches, adapted with permission [10].

Fourier transform infrared (FTIR) spectra were acquired using an Alpha II Bruker Fourier transform infrared (FTIR) instrument throughout the 4000–400 cm spectral region. The ATR technique was used for analytical reasons. The Fourier transform infrared (FTIR) spectra of potato starch and starch phthalate were acquired by analyzing samples prepared in potassium bromide (KBr) discs using a BRUKER FTIR apparatus produced in Tokyo, Japan. The samples were processed via a hydrostatic press within a pressure range of 6–8 tones, creating KBr discs [45]. Fourier transform infrared (FTIR) analysis identifies functional groups in raw materials and products [46]—the IR absorption spectrum per reference spectrum 1411 as sodium salt [35]. Figure 5 below displays the analysis results of native sago starch using Fourier transform infrared spectroscopy. Native sago starch will be compared with the results of sodium starch glycolate from sago starch to see the differences in functional groups.

### 3.5. Scanning Electron Microscopy (SEM) Analysis

**Figure 6:**
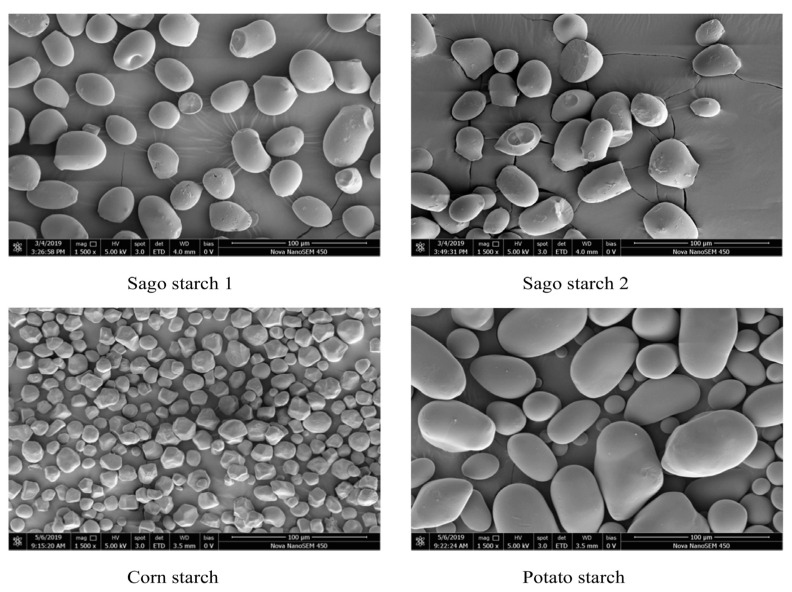
Scanning electron microscopy (SEM) images of native sago starch, corn starch, and potato starch at 1500× magnification, adapted with permission [10].

The scanning electron microscopy (SEM) method was applied to collect and analyze the surface morphology of granules. The scanning electron microscopy (SEM) instrument used for this purpose was the JSM-7200FLV model manufactured by JOEL, located in Peabody, MA, USA. The voltage applied during the imaging process was set at 5 kV. The sample was uniformly spread over a small layer on the surface of double-sided sticky tape attached to the scanning electron microscopy (SEM) stubs. The samples were subjected to a platinum coating procedure in an argon environment using the Denton Desk V TSC sputter coater (Denton Vacuum, Moorestown, NJ, USA) [47] prior to recording the surface morphology. The current research used Zeiss DSM 950 equipment (ZEISS, Oberkochen, Germany) manufactured in Germany to conduct scanning electron microscopy (SEM) analysis. The study used secondary electron signals (SE) and backscatter signals (BSE) with a resolution of 3 nm. In anticipation of the examination, a thin coating of gold was administered over the samples to augment their electrical conductivity [48]. Figure 6 below shows the morphology results of native sago starch, corn starch and potato starch.

### 3.6. Differential Scanning Calorimetry (DSC) Analysis

**Figure 7:**
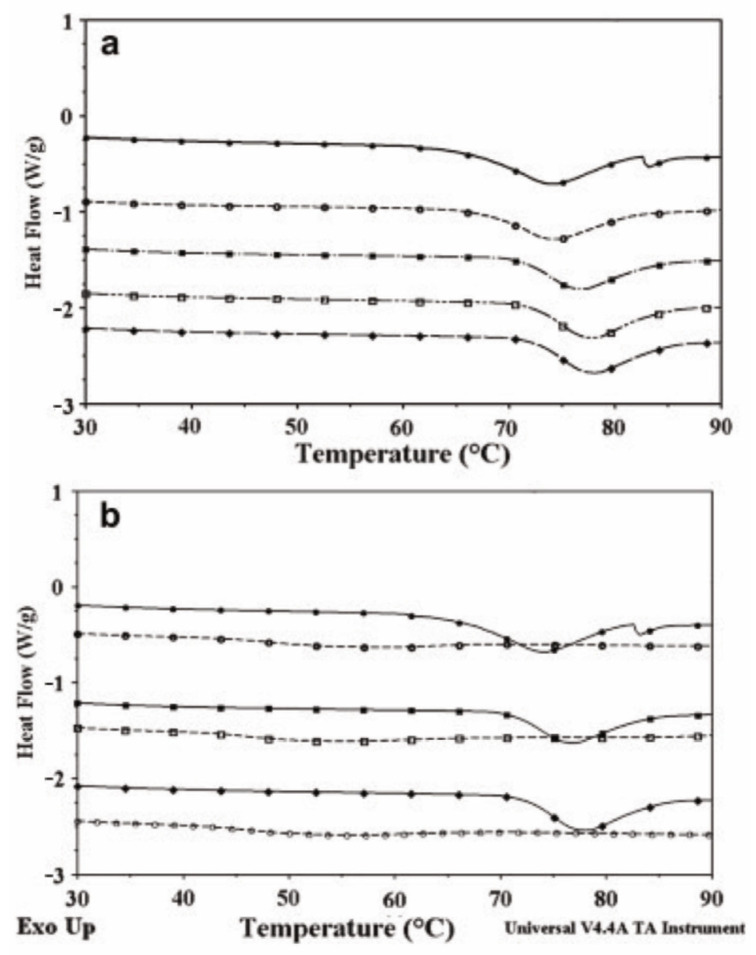
(**a**) Differential scanning calorimetry (DSC) curves (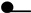: Native sago starch. 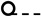: 6 h hydrolysis. 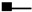: 12 h hydrolysis. 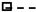: 18 h hydrolysis. 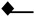: 24 h hydrolysis). (**b**) DSC thermograms of native (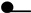), 12 h (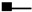), and 24 h (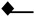) The acid-treated sago starches were evaluated for their retrogradation tendency both immediately and after being stored at 4 °C for 7 days (shown by dashed lines with empty symbols).adapted with permission [7].

The differential scanning calorimetry (DSC) thermogram of the films was acquired using a differential scanning calorimetry (DSC) Q100 instrument produced by TA Instruments, situated in New Castle, DE, USA. Before commencing the investigation, the films were conditioned at a controlled temperature of 23 ± 2 °C and a relative humidity of 50 ± 10% for 48 h. A 5 mg sample was treated to a heating process, commencing at ambient temperature and gradually increasing to 200 °C. The heating procedure was implemented with a temperature increase of 10 °C per minute, following the methods described by Thanakkasaranee et al. [49]. Nitrogen gas as a purging agent was implemented at a 50 mL/min flow rate. The report underwent a minimum of three iterations. The differential scanning calorimetry (DSC) instrument underwent calibration using ultrapure indium with a purity level of 99.999%. Indium has a melting point of 156.6 °C and a heat capacity of 28.54 J·g^−1^. This calibration method was documented in reference [50]. Differential scanning calorimetry (DSC) was performed using a Perkin Elmer Pyris 1 instrument (Perkin Elmer, Shelton, CT, USA). A 1:1 ratio of samples to distilled water was used. An aluminum pan was used to enclose the object, which was afterward sealed, and the measurement of its weight was duly documented. A vacant aluminum pan was used as a point of reference. The sealed pan underwent a temperature increase from 10 °C to 100 °C at a constant rate of 5 °C per minute [51]. Figure 7 below displays the results of the native sago starch and acid modified sago starches thermogram graph using differential scanning calorimetry (DSC).

### 3.7. Pasting Properties

Starch paste characteristics were measured using a Rapid Visco Analyzer (RVA-4, Newport Scientific, Warriewood, Australia). A quantity of 3.0 g of starch (on a dry basis) was diluted with distilled water until a consistent weight of 28.0 g was obtained in the aluminum container. The heating and cooling profile adhered to the prescribed guidelines [52]. The temperature was first maintained at 50 °C for 1 min, then raised to 95 °C at a rate of 12 °C per minute and held for 2.5 min before being lowered to 50 °C at the same rate. The starch paste was maintained at 50 degrees Celsius for 1.5 min before the pasting parameters. After acquiring the paste profiles, the viscosity parameters were determined in the following manner: the term “peak viscosity” (PV) refers to the highest level of viscosity observed during the process of heating, taking into account both the viscosity increase and the subsequent decrease caused by granule breakage.

On the other hand, “trough viscosity” (TV) represents the lowest viscosity level observed after granules collapsing. The “breakdown viscosity” (BV) is calculated as the difference between peak viscosity (PV) and trough viscosity (TV). The final viscosity (FV) refers to the viscosity seen after the conclusion of the test, which aligns with the completion of the cooling and temperature maintenance phases. During this stage, the molecules undergo reassociation, resulting in a rise in viscosity. Setback viscosity (SB) can be defined as the disparity between the final viscosity (FV) and the target viscosity (TV). The pasting temperature (PT) refers to the temperature at which the granules initiate swelling due to water absorption.

On the other hand, peak time (Pt) denotes the specific moment at which peak viscosity (PV) becomes apparent [53,54,55]. Table 3 shows the results of pasting properties of native sago starch. Next, research will be carried out to see the differences between native and modified sago.

### 3.8. Bulk and Tapped Densities

The determination of bulk density (ρ0) and tapped density (ρt) of the excipients were determined with the help of a tapped density analyzer (JV2000; Dr. Schleuninger Pharmaton AG, Solothurn, Germany). A glass cylinder with a volume of 250 mL was used to contain a powder sample weighing 100 g. The cylinder was then positioned on the tapping density tester, and the volume of the powder in its loose state was measured and documented. The cylinder was subjected to 1000 taps at a consistent volume, and the volume resulting from each tap was documented. The assessment of bulk and tapped densities was performed by analyzing the ratio of weight to volume. The experimental protocol was performed in triplicate [56,57]. The Carr index was calculated based on the measurements of bulk density and tap density [26].

(10) $$\mathrm{Carr}\;\mathrm{index}=1-\frac{bulk\;density}{tap\;density}$$

### 3.9. Surface Area, Pore Volume, and Pore Size

The Quantachrome Autosorb 1-C (model AS1-OT, Quantachrome Instruments, Boynton Beach, FL, USA) instrument determined the surface area, pore size, and pore volume. The measurement was conducted by subjecting the sample to N_2_ adsorption and using the BJH (Barrett–Joyner–Halenda) technique. Each measurement was duplicated [58]. The measured surface area of Glycolys is 0.24 m^2^/g, Primojel has a surface area of 0.185 m^2^/g, and Tablo has a surface area of 0.335 m^2^/g [25].

### 3.10. Hydration Capacity

The methodology described in reference [59] was used. A 1 g sample was distributed evenly among four 15 mL polypropylene centrifuge tubes. Each tube was then sealed by adding 10 mL of distilled water from a 10 mL graduated cylinder. The ingredients were subjected to agitation on a vortex mixer for 2 min. Subsequently, the mixture remains undisturbed for 10 min, after which it is promptly subjected to centrifugation. The supernatant was decanted with caution, and afterward, the sediment was measured in weight. The hydration capacity was determined by calculating the ratio of the sediment’s weight to the dry sample’s weight [59].

### 3.11. Water Solubility and Swelling Power

The assessment of the samples’ aqueous solubility and swelling capacity included the introduction of 0.1 g of each sample into pre-weighed centrifuge tubes holding 10 mL of water with a concentration of 1% *w*/*v*. The contents were then thoroughly mixed for 1 min using a vortex mixer. The tube was subjected to a 10 min incubation period, followed by centrifugation at a speed of 3000 revolutions per minute (rpm) for 15 min. The liquid portion was carefully moved to a crucible that had been weighed beforehand and then subjected to a drying process at 120 °C until a consistent weight was achieved [60]. The solubility was determined by calculating the weight of the dried residue, whereas the swelling power was determined by calculating the weight of the precipitated paste [61].

### 3.12. Action Procedure

SSG, or sodium starch glycolate, is a carboxymethyl starch (CMS) with a relatively low degree of substitution (DS). It possesses a granular morphology and exhibits a quick and broad swelling behavior while forming minimum gel structures. This process entails the quick absorption of moisture, leading to significant expansion of the granules and thus facilitating rapid and even disintegration (see Figure 6) [17]. Formulations often employ percentages ranging from 2% to 8%, with an ideal concentration of around 4%. However, it is worth noting that a concentration of 2% is frequently seen as satisfactory. The phenomenon of disintegration is attributed to the quick uptake of water, which is subsequently accompanied by a substantial and rapid increase in volume [25]. The optimal concentration range for achieving desired results is between 4% and 6%. When the concentration exceeds 8%, the gelation process occurs, which subsequently affects the viscosity and results in considerably prolonged disintegration periods [62]. The gel has undergone prolonged exposure to water. Elevated concentrations of certain substances can lead to gelation and the subsequent degradation of perishable items. The sodium starch glycolate’s (SSG) operational mechanism was identified using real-time, high-resolution magnetic resonance imaging (MRI). The determination of the functioning mechanism of sodium starch glycolate (SSG) involves an examination of the tablet’s expansion direction during disintegration. The phenomenon of swelling in self-supporting gels has been documented in different grades of sodium starch glycolate (SSG), with expansion occurring in many directions [63].

### 3.13. Biopharmaceutical Quality

The pH of the medium has an impact on the functioning of sodium starch glycolate (SSG). The sodium starch glycolate (SSG) molecule experiences hydration because of the interaction between its anionic carboxyl group and water molecules. The procedure involves the introduction of anionic groups by grafting. The carboxymethyl starch (CMS) method is commonly used in an organic phase to attain substantial yields and a notable degree of substitution (DS) [64,65,66,67]. Sodium starch glycolate is a carboxymethylated starch derived from potatoes that is commercially accessible and exhibits significant superdisintegrant properties [25]. The usage of anionic carboxymethyl starch (CMS) as a medicinal excipient that exhibits responsiveness to variations in pH has been observed. A partially amorphous starch excipient was successfully synthesized using the carboxymethylation of starch with a relatively low degree of substitution (DS) in an aqueous environment. When exposed to stomach acidity, tablets containing carboxymethylation starch will undergo the formation of an outer layer with increased density due to the protonation of carboxylic groups. The layer serves as a protective barrier, effectively inhibiting the penetration of the acidic environment into the tablet [68]. The attainment of a neutral state of polymer occurs at low pH conditions, resulting in a reduced affinity for water.

**Figure 8:**
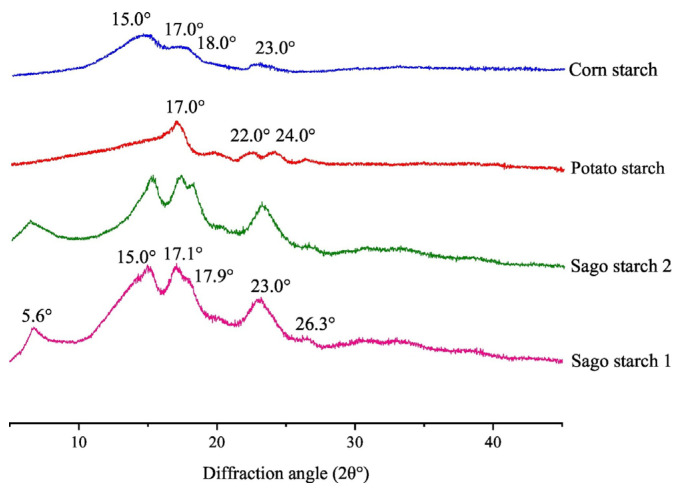
X-ray diffraction patterns (XRD) of starches, adapted with permission [10].

In contrast to the intestinal media with phosphate support and a pH of 6.8, the simulated gastric media with 0.1 N HCl and a pH of 1 exhibit more significant swelling in the sodium starch glycolate (SSG) [69]. Figure 8 displays the graphic results of native sago starch to see the crystallinity of native sago starch. The chromatogram shows the crystalline area and the amorphous area of sago starch.

### 3.14. Molecular Characteristics

The characterization of the amount of substitution is necessary due to the functional influence of carboxymethyl assembly. According to the United States Pharmacopeia (USP), the sodium content in sodium starch glycolate (SSG) falls within the range of 2.8% to 4.2%, but the degree of replacement remains unspecified. Nevertheless, previous studies have documented substitution levels ranging from 0.23 to 0.32. The rapid disintegration of tablets can be attributed to the hydration and swelling of sodium starch glycolate (SSG), which is influenced by the amount of substitution. An increase in swelling and water intake is detected as the substitution level rises from 0.20 to 0.29, while the opposite effects are shown at higher substitution levels. A preferable range of replacement values, namely between 0.28 and 0.29, was reported to enhance the speed and efficacy of dissolving aspirin pills. The interaction between medications and excipients can be enhanced by increasing the amount of substitution, as weakly essential pharmaceuticals can be absorbed into the polymer material [70]. Sodium starch glycolate (SSG) is obtained via the chemical modification of starch, including two fundamental processes: substitution, which enhances its hydrophilic properties, and cross-linking, which decreases its solubility and ability to form gels when exposed to water. Sodium starch glycolate (SSG) functions as a very effective disintegrant by rapidly swelling due to the absorption of significant quantities of water, resulting in expedited breakdown [71].

**Figure 9:**
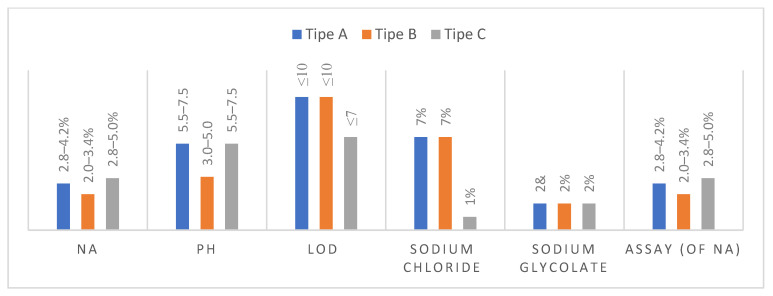
Pharmacopeia specifications for various brands of sodium starch glycolate (SSG).

In comparison to other swelling disintegrants, the enhanced swelling of sodium starch glycolate (SSG) can be attributed to its specific cross-linking mechanism. For instance, croscarmellose is cross-linked by esterification, which restricts the formation of significant gaps between the polymer chains, limiting its swelling capacity. A 25–35% increase in cross-linking results in enlarged swelling and water uptake. However, a further increase in cross-linking leads to reduced swelling and water uptake [72]. A reported optimal value for the disintegration of aspirin tablets has been seen at moderate levels of cross-linking, namely within the range of 33–35%. Sodium starch glycolate is a kind of potato starch that is chemically modified and cross-linked. The substance may be obtained from several manufacturers, each offering it under different brand names, such as Explotab, Primojel, and Vi-vastar [73]. The chemical composition and physical characteristics of Explotab and Primojel have been thoroughly examined in commercial assessments, revealing subtle differences. The bulk density of the 4% *w*/*v* aqueous dispersion is measured to be 0.756 g/cc, while its viscosity is determined to be 200 cP. According to Iram et al. (2017) [21], it has been observed that sodium starch glycolate (SSG) does not undergo a melting process but rather undergoes charring at an estimated temperature of 200 °C. The study of molecular properties is a fundamental aspect of chemistry. It involves investigating and analyzing various characteristics and behaviors exhibited by molecules. Table 4 and Figure 9 display the documented specifications involved in producing sodium starch glycolates (SSGs) of various brands or types.

Following Table 4, the further specifications of sodium starch glycolate (SSG) of various brands or types according to Pharmacopeia specifications are shown in the figure below.

### 3.15. Dynamic Vapor Adsorption (DVS) Characteristics

In recent years, there has been a rise in the utilization of automated dynamic vapor sorption (DVS) methodologies. The initial documentation of a manual dynamic vapor sorption (DVS) system dates back over 30 years [74]. The moisture absorption properties of sodium starch glycolate were evaluated using the dynamic vapor sorption (DVS) method. The findings were compared to the data obtained from potato starch, microcrystalline cellulose, and pregelatinized starch (PGS). Sodium starch glycolates (SSG) have a much higher mass augmentation when exposed to a relative humidity of 90% compared to other excipients derived from anhydrous glucose. However, potato starch and the modified starches have comparable sorption capabilities over the whole period. The relative humidity ranges from 10% to 70%. The findings indicate that the sorption capacity within the ranges from 10% to 70% relative humidity remains largely unaffected by the particular methods of cross-linking and sodium carboxymethylation used in sodium starch glycolates, as well as the process of gelatinization in pregelatinized starch (PGS). Moreover, the augmented disintegrant capabilities exhibited in sodium starch glycolates probably stem from an intricate interplay between water molecules and the starch structure, surpassing the number of hydration sites that are accessible [18].

## 4. Sodium Starch Glycolate (SSG) for Superdisintegration, Derived from Sago Starch

### 4.1. Formulation Evaluation

**Figure 10:**
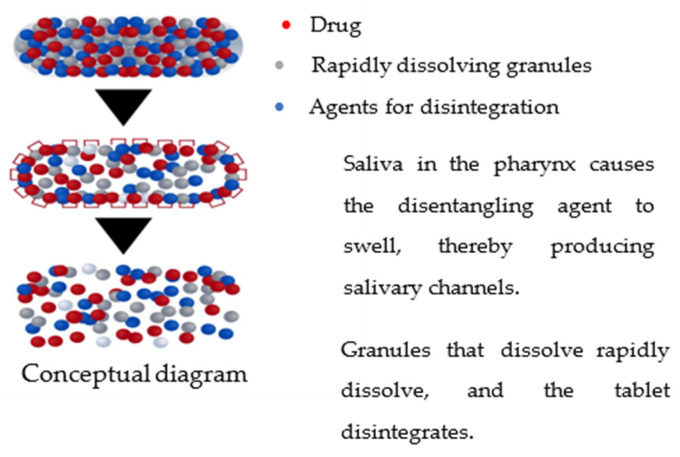
Conceptual mechanism of FDT disintegrating [75], note: red is the drug, gray is rapidly dissolving granules, and blue is agents for disintegration.

**Figure 11:**
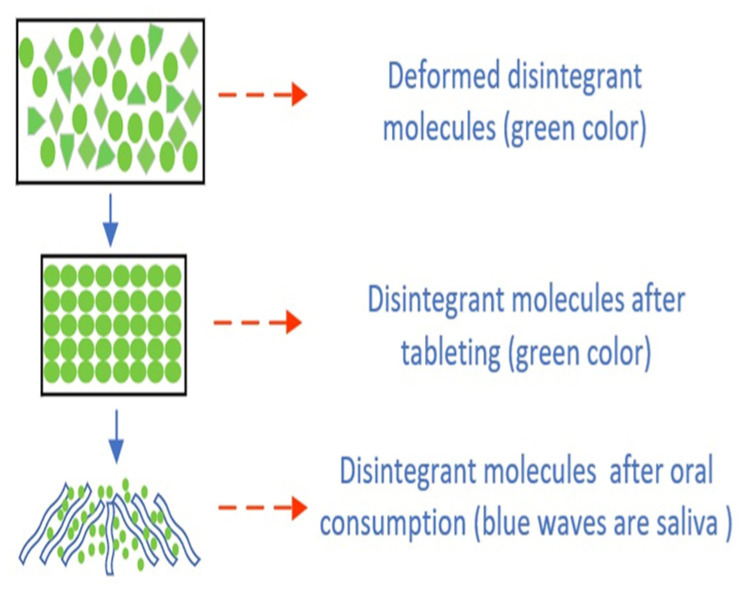
Action mechanism of a mouth-dissolving tablet containing sodium starch glycolate (SSG) as a superdisintegrant, note ● green: drug, and ◊ green: solvent and other excipient.

The percentage range of sodium starch glycolate (SSG) in medication formulations varies from 2% to 8% *w*/*w*, as reported in reference [25]. Increased concentrations of sodium starch glycolate (SSG) at 2% and 4% weight/weight (*w*/*w*) in paracetamol led to decreased disintegration periods, with disintegration occurring within 1 min. Conversely, lower concentrations of sodium starch glycolate (SSG) at 0.25%, 0.5%, and 1% *w*/*w* resulted in prolonged disintegration durations and altered decay times, ranging from 60 min to 2–15 min. Extended disintegration durations are seen when the tablet weight contains more significant quantities of sodium starch glycolate (SSG), surpassing 8% [70]. Regardless of the API’s solubility, the drug must be taken orally. The hindrance of water entry into the formulation can be ascribed to establishing a thick layer [21]. Figure 10 and Figure 11 below explains the conceptual mechanism of FDT disintegration in the mouth.

### 4.2. Powder Compression Qualities

Values for the degree of substitution (DS) between 0.23 and 0.32 have been found in previous investigations [69]. The level of replacement has an impact on the hydration and swelling characteristics of sodium starch glycolate (SSG). The study reported an apparent swelling and water intake increase when the substitution level increased from 0.20 to 0.29. Conversely, an opposing impact was detected when introducing higher substitution levels [76]. An elevated substitution level might lead to an augmentation in drug–excipient interactions for a weak primary agent due to the possibility of the drug being adsorbed into the polymer [77]. The effects of 0.17 and 30 mm/s pressure rates on Explotab, Primojel, and Vivastar P were studied. According to the data, the three available materials respond differently to pressure and compacting [12].

Explotab and Primojel exhibit comparable compatibility, whereas Vivastar P has the lowest level of compatibility. The observed behavior did not manifest in the compressibility characteristics of the powders, as both Vivastar P and Explotab exhibited similar performance. The materials underwent analysis using X-ray diffraction (XRD), scanning electron microscopy (SEM), Carr’s compressibility index, and swelling volume measurement [32]. Regarding the material properties, all the objects exhibited similar levels of swelling when exposed to water. According to characterization investigations, Vivastar P has been identified as a distinct material in terms of its moisture content, crystalline order, and particle topography compared to Primojel and Explotab [12].

### 4.3. The Property of Enhancing Solubility

The compound sodium starch glycolate (SSG) demonstrates a lack of solubility in water, whereas it displays limited solubility in 95% ethanol. This substance exhibits a notable level of insolubility in aqueous solutions. At a concentration of 2% *w*/*v*, sodium starch glycolate demonstrates dispersion in cold water and subsequently forms a layer with high hydration upon settling [78]. Most pharmaceutical substances exhibit low water solubility and require an excipient to enhance their solubility. Several hydrophilic swellable polymers, including sodium carboxymethyl cellulose (Na-CMC), sodium starch glycolate (SSG), and pregelatinized starch (PGS), have been included in solid dispersion formulations to enhance the dissolving characteristics of medications with limited solubility [79].

### 4.4. The Impact of Self-Emulsifying Drug Delivery Systems (Sodium Starch Glycolate—SSG) on the Stability of Dosage Forms

Chemical instability in solid dosage forms arises due to the medication’s and excipients’ interaction. Solid dosage forms exhibit lower stability than active pharmaceutical ingredients (APIs). A chemical reaction occurs when an excipient functions as a catalyst and directly interacts with the drug molecule [80]. The microenvironment experiences a pH alteration that results in an enhancement in the rate of chemical reactions. The chemical instability of solid dosage forms can be attributed to several reactions, including hydrolysis, dehydration, isomerization, elimination, cyclization, oxidation, and photodegradation. Sodium starch glycolate may include reactive impurities such as monochloroacetate, nitrites, and nitrates. Electrostatic interactions give rise to an observed instance of incompatibility in the adsorption of weakly essential medications and their salts.

Furthermore, the leftover monochloroacetate has the potential to conduct an SN2 nucleophilic reaction, as shown by previous research [81]. Table 5 below shows several uses of sodium starch glycolate in various medicinal preparations. Sodium starch glycolate is one of the most widely used excipients in every drug formulation.

## 5. Conclusions and Prospects

Sodium starch glycolate is the excipient that is most often used among all available excipients. Pharmaceutical formulations often use this substance as a superdisintegrant. The efficacy of sodium starch glycolate as a disintegrant is influenced by its physical qualities, which are determined by factors such as the level of cross-linking, degree of carboxymethylation, and purity. Sago starch is considered to be a promising source of natural macromolecules. Indonesia is among the leading global producers of sago, with a position among the top three. Compared to other starches, sago starch is characterized by its comparatively low cost.

Moreover, sago starch is listed in the handbook of excipients as a prospective alternative raw material for excipients in tablet formulations. Sago starch shares many characteristics with cassava, maize, and potato starches. Compared to pea and corn starch, sago starch is more swellable and less enzymatically digestible.

Both the sodium starch glycolate (SSG) concentration and the polymer particles’ size influence the disintegration duration of a formulation utilized. Superdisintegrants have been used in several applications, such as oral disintegration tablets, rapid dispersible tablets, capsules, mouth-dissolving films, etc. Examination and analysis of the degree of substitution (DS) with atomic absorption spectroscopy (AAS) and sodium chloride (NaCl) Measurement with titration of the elemental composition of sodium starch glycolate (SSG) particles using energy-dispersive X-ray spectroscopy or scanning electron microscopy (SEM), pasting properties, and Fourier transform infrared (FTIR).

There are 5.5 million hectares of potential sago land in Indonesia. However, only 5% of it is now being used. Thus, the potential development of sago needs to be explored, and novel development and application of sago starch should be used to create superdisintegrant tablets or capsules.

## Figures and Tables

**Table 1:** Starch chemical composition [10].

Starch	Moisture (%)	Total Starch (%)	Proteins (%)	Lipid (%)	Ash (%)	Amylose (%)	RS (%)
Sago	13.33 ± 0.04 ^c^	87.12 ± 0.22 ^b^	0.17 ± 0.00 ^c^	87.12 ± 0.22 ^b^	0.08 ± 0.00 ^d^	26.11 ± 0.21 ^a^	34.62 ± 0.40 ^c^
Corn	11.72 ± 0.03 ^d^	93.76 ± 0.77 ^a^	0.33 ± 0.03 ^b^	0.62 ± 0.09 ^a^	0.21 ± 0.02 ^b^	13.35 ± 0.24 ^c^	4.00 ± 0.18 ^d^
Potato	19.28 ± 0.05 ^a^	84.82 ± 0.41 ^c^	0.87 ± 0.00 ^a^	0.66 ± 0.01 ^a^	0.28 ± 0.01 ^a^	11.67 ± 0.19 ^d^	60.43 ± 0.47 ^a^

The results represent the means ± standard deviations of triplicate analyses. Values with distinct letters within the same column show a significant difference, as indicated by a *p*-value less than 0.05.

**Table 2:** Sodium starch glycolate (SSG) of various brands.

Excipient Sodium Starch Glycolate (SSG)	pH Value	Source	Manufacturing Process	Main Application
EXPLOTAB^®^—JRS Pharma, Rosenberg, Germany	5.5–7.5	Potato starch	Explotab is produced by cross-linking and carboxymethylation with a hot plate or reactor.	Rapid and substantial swelling superdisintegrant for tablet and capsule formulations.
				Particularly in the case of water-insoluble active compounds and tablet matrices, they exhibit elevated pH levels.
EXPLOTAB^®^ CLV—JRS Pharma, Rosenberg, Germany	5.5–7.5	Potato starch	Explotab is produced by cross-linking and carboxymethylation with a hot plate or reactor.	The particular grade exhibits an elevated quantity of cross-linkages.
				Particularly well suited for wet granulation applications.
EXPLOTAB^®^ Low pH—JRS Pharma, Rosenberg, Germany	3.0–5.0	Potato starch	Explotab is produced by cross-linking and carboxymethylation with a hot plate or reactor.	A grade characterized by a low pH value.
				The product conforms to the specifications of the European Pharmacopoeia (Ph. Eur) and the National Formulary (NF) for type B typography.
GLYCOLYS^®^—Roquette Freres 1 Rue de la Haute Loge LESTREM—France	5.5–7.5	Potato starch	Glycolys^®^ is produced by cross-linking and carboxymethylation with a hot plate or reactor.	A superdisintegrant exhibiting a rapid and substantial swelling capacity is advantageous in developing tablet and capsule formulations.
GLYCOLYS^®^ LV—Roquette Freres 1 Rue de la Haute Loge LESTREM—France	5.5–7.5	Potato starch	Glycolys^®^ LV is produced by cross-linking and carboxymethylation with a hot plate or reactor.	For high-shear granulation addition in the intragranular phase.
GLYCOLYS^®^ Low pH—Roquette Freres 1 Rue de la Haute Loge LESTREM—France	3.0–5.0	Potato starch	Glycolys^®^ low pH is produced by cross-linking and carboxymethylation with a hot plate or reactor.	For acidic drugs.
Primojel^®^—DFE PHARMA, Germany	5.5–7.5	Potato starch	Potato starch is cross-linked and carboxy-methylated to produce this substance. It is a white substance that flows freely.	Primojel^®^ can function as a dissolution enhancer in greater concentrations. This ingredient is exceptionally efficacious when used intragranularly and/or extragranularly in granular formulations.
VIVASTAR^®^ P—JRS Pharma, Rosen-berg, Germany	5.5–7.5	Potato starch	A superdisintegrant for tablets and other solid oral dosage forms can be manufactured by utilizing potato starch through the carboxymethylation and cross-linking processes.	A superdisintegrant exhibiting a rapid and substantial swelling capacity is advantageous for developing tablet and capsule formulations, particularly in active ingredients with low solubility in water and tablet matrices exhibiting elevated pH levels.
VIVASTAR^®^ PSF—JRS Pharma, Rosenberg, Germany	5.5–7.5	Potato starch	The superdisintegrant is produced by carboxymethylation and cross-linking of potato starch.	The particular grade has a significantly reduced concentration of methanol. It is particularly well suited for active pharmaceutical ingredients (APIs) sensitive to alcohol and moisture.
VIVASTAR^®^ P 1000 SF	5.5–7.5	Potato starch	Potato starch is carboxymethylated and cross-linked to create the superdisintegrant.	Superdisintegrants with varying viscosities, classified as low, medium, or high, can produce transparent gels when exposed to water, following type C specifications outlined in the European Pharmacopoeia (Ph.Eur.). This is under type A specifications outlined in Japanese Pharmacopeia (JP) and National Formulary (NF) standards.
VIVASTAR^®^ P 3500—JRS Pharma, Rosenberg, Germany				
VIVASTAR^®^ P 5000—JRS Pharma, Rosenberg, Germany				

**Table 3:** Pasting properties of starches [10].

Starch	Pasting Parameters					
	PT (°C)	PV (cP)	TV (cP)	BD (cP)	FV (cP)	SB (cP)
Native Sago	80.2 ± 0.4 ^a^	515.7 ± 9.5 ^d^	221.7 ± 3.1 ^d^	294.0 ± 6.6 ^c^	464.2 ± 6.0 ^d^	242.5 ± 3.2 ^b^
Sodium Starch Glycolate (SSG) Sago	-	-	-	-	-	-

Results are means ± standard deviations of duplicate analysis. Values with the different letters in the same column are significantly different (*p* < 0.05). PT: pasting temperature; PV: peak viscosity; TV: trough viscosity; BD: breakdown (PV—TV); FV: final viscosity; SB: setback.

**Table 4:** Pharmacopeia specifications for various brands of sodium starch glycolate (SSG).

Tests	Type A	Type B	Type C
Definition	The substance is cross-linked, partly O-carboxymethylated potato starch in sodium salt form	The substance is cross-linked, partly O-carboxymethylated potato starch in sodium salt form	One kind of cross-linked starch’s sodium salt, which has undergone physical dehydration and partial O-carboxymethylation
Size	30–100 µm	30–100 µm	30–100 µm
Identification	The infrared absorption spectrum, following the reference spectrum	Iodine-blue color	(1) The formation of a white precipitate of K-antimonate (2) The addition of magnesium to uranyl acetate results in the formation of a yellow precipitate

**Table 5:** Top medications with this excipient.

Active Pharmaceutical Ingredient (API)	Tablets Preparation	Reference
Loratadine	OT	[82]
Ramipril	FMDT	[83]
Ondansetron	FDT	[84]
Ibuprofen	Crystal engineering to improve pharmaceutical performance	[85]
Salbutamol sulfate, cetirizine hydrochloride in combination	FDT	[86]
Almotriptan malate	MDF	[87]
Nortriptyline hydrochloride	FDT	[88]
Flutamide	OT	[89]
Losartan potassium	FDT	[87]
Valacyclovir hydrochloride	SRT	[90]
Sildenafil citrate nanocrystals	FDT	[91]
Valsartan	BT	[92]
Simvastatin	FDT	[93]
Salbutamol sulfate	FDT	[94]
Cefixime trihydrate	FDT	[95]
Amlodipine besylate and Atorvastatin calcium	FDT	[96]
Venlafaxine hydrochloride	FDT	[97]
Celfdinir solid dispersion	FDT	[98]
Telmisartan	FDT	[99]
Furosemide	FDT	[100]
Tizanidine hydrochloride	FDT	[101]
Doxylamine succinate	OT	[102]
Amlodipine besylate	MDT	[103]
Metoclopramide hydrochloride	SRT	[62]
Nifedipine	ST	[63]
Aloe vera gel	FDT	[104]

Note: The various kinds of tablet preparations are as follows: FMDT = fast mouth-dissolving tablets; FDT = fast disintegrating tablets; OT = orodispersible tablet; MDF = mouth-dissolving film; BT = bilayer tablets; SRT = sustained release tablet; MDT = mouth dispersible tablet; ST = sublingual tablets.

## Data Availability

Suggested Data Availability Statements are available in section “MDPI Research Data Policies” at https://www.mdpi.com/ethics (accessed on 16 November 2023).
